# ICTV Virus Taxonomy Profile: *Nyamiviridae*

**DOI:** 10.1099/jgv.0.000973

**Published:** 2017-11-09

**Authors:** Ralf G. Dietzgen, Elodie Ghedin, Dàohóng Jiāng, Jens H. Kuhn, Timothy Song, Nikos Vasilakis, David Wang, ICTV Report Consortium

**Affiliations:** ^1^​ Queensland Alliance for Agriculture and Food Innovation, University of Queensland, St. Lucia, Queensland, Australia; ^2^​ Center for Genomics and Systems Biology, Department of Biology, New York University, New York, NY, USA; ^3^​ State Key Laboratory of Agricultural Microbiology, The Provincial Key Lab of Plant Pathology of Húběi Province, College of Plant Science and Technology, Huázhōng Agricultural University, Wuhan, PR China; ^4^​ Integrated Research Facility at Fort Detrick, National Institute of Allergy and Infectious Diseases, National Institutes of Health, Frederick, MD, USA; ^5^​ Center for Biodefense and Emerging Infectious Diseases, Department of Pathology, The University of Texas Medical Branch, Galveston, TX, USA; ^6^​ Departments of Molecular Microbiology and Pathology and Immunology, Washington University School of Medicine, St. Louis, MO, USA

**Keywords:** *Nyamiviridae*, ICTV report, taxonomy, Nyamanini virus, Midway virus, soybean cyst nematode virus 1

## Abstract

The *Nyamiviridae* is a family of viruses with unsegmented, negative-sense RNA genomes of 11.3–12.2 kb that produce enveloped, spherical virions. Viruses of the genus *Nyavirus* are tick-borne and some also infect birds. Other nyamiviruses infecting parasitoid wasps and plant parasitic nematodes have been classified into the genera *Peropuvirus* and *Socyvirus,* respectively. This is a summary of the current International Committee on Taxonomy of Viruses (ICTV) Report on the taxonomy of *Nyamiviridae,* which is available at www.ictv.global/report/nyamiviridae.

## Abbreviations

G, glycoprotein; L, large protein; M, matrix protein; N, nucleocapsid; P, phosphoprotein.

## Virion

**Fig. 1.**
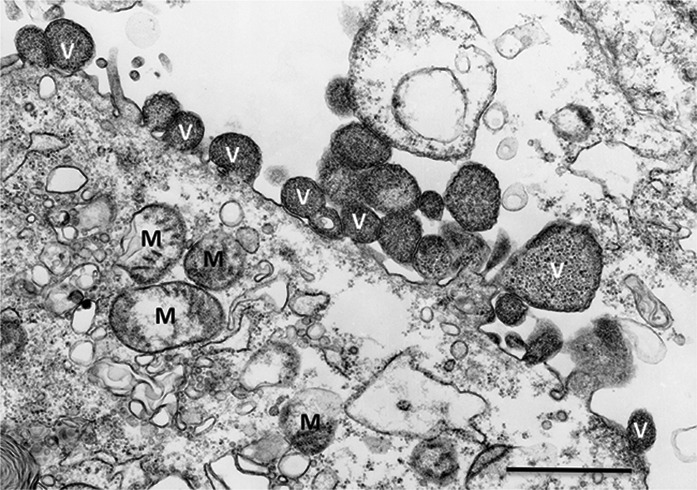
Transmission electron micrograph of Vero E6 cells infected with Sierra Nevada virus. High magnification of virions (V) budding from the cell surface. Mitochondria (M) are indicated for reference. Scale bar=1 µm. (Contributed by Dr Vsevolod Popov, Department of Pathology, Center for Biodefense and Emerging Infectious Diseases, University of Texas Medical Branch, Galveston, TX, USA).

Virions are enveloped and spherical with a diameter of 100–130 nm (Table 1, Fig. 1) [1].

**Table 1.** Characteristics of the family *Nyamiviridae*

**Typical member**	Nyamanini virus (FJ554526), species *Nyamanini nyavirus*, genus *Nyavirus*
Virion	Enveloped, spherical particles, approximately 100–130 nm in diameter
Genome	Negative-sense, single-stranded, unsegmented RNA of 11.3–12.2 kb
Replication	Nuclear: the RNA-dependent RNA polymerase engages with ribonucleoprotein at the genome 3′ end
Translation	Individual putatively polyadenylated mRNAs are translated in the cytoplasm
Host range	Invertebrates: ticks, parasitoid wasps, nematodes; vertebrates: land- and seabirds
Taxonomy	The genera *Nyavirus*, *Peropuvirus* and *Socyvirus* include five species

## Genome

**Fig. 2.**
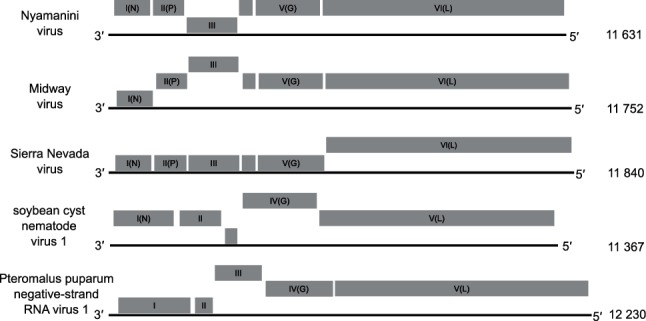
Genome organization of viruses in the family *Nyamiviridae*. Boxes indicate the position and length of each ORF (numbered consecutively I–VI or I–V), with boxes that are in the same vertical position relative to the solid line (representing the genome) indicating ORFs that are in the same reading frame. Putative nucleocapsid (N) protein, phospho- (P) protein, glycoprotein (G) and large (L) protein ORFs are indicated. The number of nucleotides comprising each genome is indicated.

Nyamivirus negative-sense single-stranded RNA genomes range from 11.3 to 12.2 kb (Fig. 2). All known nyamiviruses have unsegmented genomes with five or six ORFs that encode the structural proteins. Among them are the nucleocapsid (N) protein, glycoprotein (G) and large (L) protein, which are identified based on sequence similarity and structural properties shared with mononegavirus homologues. Functions of the other encoded proteins are largely unknown but may be those of matrix (M) and polymerase cofactor [phospho- (P)] proteins.

## Replication

Knowledge about nyamivirus replication is limited. Nyamanini virus (genus *Nyavirus*) replicates in the nucleus of cells [2] by a complex consisting of the viral nucleoprotein (N), polymerase cofactor (P) and the large (L) protein which form an active RNA-dependent RNA polymerase that engages with the ribonucleoprotein at the 3′ end of the genome. mRNAs are transcribed processively from each gene (3′ to 5′). Nyaviral genes are separated by conserved motifs for transcription initiation and termination. Encoded core proteins, polymerase activity, nuclear replication and particle formation appear to be similar to members of the mononegaviral families *Filoviridae* and *Bornaviridae*.

## Pathogenesis

Some nyavirus isolates cause cytopathic effects in tissue culture. Nyamanini virus causes plaques in duck embryo and rhesus monkey kidney cells and cytopathic effects in BHK-21 cells. Midway virus is cytopathic for BHK-21 cells and produces plaques in Vero cells.

## Taxonomy

The *Nyamiviridae* family includes the three genera *Nyavirus*, *Peropuvirus* and *Socyvirus*. Nyamanini virus and Midway virus (genus *Nyavirus*) are tick-borne and infect birds, but it is unclear if tick-borne Sierra Nevada virus (genus *Nyavirus*) can also infect birds [2–5]. Soybean cyst nematode virus 1 (genus *Socyvirus*) infects plant parasitic nematodes [6], while Pteromalus puparum negative-strand RNA virus 1 (genus *Peropuvirus*) was isolated from parasitoid wasps [7]. Viruses assigned to each genus form a monophyletic clade on phylogenetic analysis of L protein sequences, although bootstrap support is weak. These viruses have a similar genomic organization, including the number and locations of genes identified by homology with those of other mononegaviruses (Fig. 2). Given the extent of divergence of viruses in the *Peropuvirus* genus from viruses in the rest of the family, this genus may, in the future, need to be reclassified outwith the family *Nyamiviridae*.

## Resources

Full ICTV Online (10th) Report: www.ictv.global/report/nyamiviridae.
